# Simulation‐Based Comparison of Standard Versus Rotational Laryngeal Mask Airway Insertion Techniques in Novice Anesthesiologists: A Randomized Controlled Crossover Trial

**DOI:** 10.1155/anrp/7850095

**Published:** 2025-12-31

**Authors:** Kumar D., Kala C.

**Affiliations:** ^1^ Department of Anesthesia, Aga Khan University Hospital, Karachi, 74800, Pakistan, aku.edu

**Keywords:** LMA, manikin, rotational LMA technique, simulation, standard LMA technique

## Abstract

**Background:**

Anesthesia trainers frequently experienced desaturation, airway trauma, and failed intubation with novice hands‐on training for facemask and endotracheal intubation. Laryngeal mask airway (LMA) adaptation in anesthesia practice has ensured trainers’ ease in safely transforming sufficient airway management skills to develop competency among novice trainees. This study was designed to evaluate the manikin‐based standard versus rotational LMA insertion techniques to analyze insertion ease and priority LMA insertion technique among novice anesthesia trainees.

**Methods:**

Our randomized controlled crossover trial has enrolled 13 novice anesthesia trainees from multicenter hospitals. The study comprised three phases. Initially, the study participants were inquired on a preformed questionnaire for supraglottic airway device knowledge, insertion techniques, and experiences. In the second phase, trainees have undergone educational tutorial, video presentation, and hands‐on practice on a manikin for standard and rotational LMA insertion techniques. Following that, the participants were divided into two study groups to evaluate LMA insertion, 10 times each with standard and rotational in a crossover manner. The third phase of the study was also questionnaire‐based; trainees were inquired about the preferred LMA insertion technique for beginners.

**Result:**

The trainee’s prior knowledge of supraglottic airway devices and practical experience were equivocal. There were no insertion failure attempts with both standard and rotational LMA insertion techniques. The time duration for the first attempt with standard LMA insertion was significantly shorter (7.92 ± 2.43 s) compared to the time duration for the first attempt with rotational LMA insertion (11.80 ± 3.41 s) on manikin. The post–study analysis revealed trainees’ preference for the standard LMA insertion technique.

**Conclusion:**

Our study concludes that the standard LMA insertion technique is relatively easy to learn and practice on manikin compared to the rotational technique. Besides this, the novice anesthesia trainees have preferred standard LMA insertion as a priority technique in their clinical practice.

**Trial Registration:**

ClinicalTrials.gov identifier: NCT05544838

## 1. Introduction

Anesthesia training usually starts with airway management to ensure optimal oxygenation and ventilation to warrant safe anesthesia practice [1]. In an old era, the trainers were frequently experiencing desaturation, airway trauma, and failed intubation [2], with the beginners’ hands‐on training for facemask and endotracheal intubation. Laryngeal mask airway (LMA) adaptation in anesthesia practice has ensured trainers’ ease in safely transforming sufficient airway management learning skills in clinical practice [3, 4]. The trainees have also acquired competency with LMA in a short duration, especially in the early phase of anesthesia training year. The LMA has rapidly gained popularity in airway management, and it has become the first choice in an indicated elective surgical procedure [5]. The LMA is also recommended in practice guidelines for the difficult or failed airway management scenarios to rescue the patient’s airway in emergency medicine, intensive care, and prehospital setting [6, 7]. Although the LMA insertion learning curve is shorter, it requires some degree of technical skills to negotiate into oral cavity structures and to place at the optimal insertion seating position [8]. The simulation teaching in a controlled environment presents learning opportunities among trainees to develop tactile skills in anesthesia, surgery, and emergency medicine to practice critical maneuvers in a repetitive manner on manikins [9, 10]. The literature supports the benefit of simulation‐based medical education (SBME), and a recent meta‐analysis [11] on simulated airway management training revealed learners’ satisfaction with the knowledge acquisition, technical skills, and clinical outcomes in comparison with traditional learning methods. Another manikin‐based meta‐analysis evaluated insertion ease with the first‐ and second‐generation supraglottic airway devices (SADs), and the study revealed the sequential lowest insertion time with Aura, LMA, i‐gel, and air‐Q among the unskilled healthcare professionals [12, 13]. The clinical studies on human subjects have also revealed the first‐attempt success rate of 67%–90%, with the standard LMA insertion technique, compared to 86%–99%, with the rotational LMA insertion technique [7, 14, 15].

Our study aim was to evaluate the manikin‐based standard versus rotational LMA insertion techniques to analyze the insertion ease and priority LMA insertion technique among novice anesthesia trainees. The primary aim of our study was to compare the LMA insertion ease with standard and rotational insertion techniques in terms of the number of LMA insertion attempts and the LMA insertion time duration by novice anesthesiologists on a manikin. The secondary aim was to observe the impact of prior knowledge of the SADs, insertion techniques, and general and specific work experience in airway management. The tertiary aim was to determine the priority LMA insertion technique in a clinical practice among novice anesthesia trainees.

## 2. Materials and Methods

### 2.1. Study Ethics and Setting

The study was approved by the Research Ethics Committee of Aga Khan University (Approval Number 2022‐6256‐22361). This simulation study was conducted on October 2, 2021, at the Center for Innovation in Medical Education (CIME) at Aga Khan University Hospital, Karachi.

### 2.2. Airway Manikin and SAD

The Laerdal SimMan (Laerdal Medical AS, AUS) manikin was selected in our study after thorough evaluation by two experienced anesthesiologists (with insertion experience of more than 1000 times). The manikin was examined for practical adaptability, and it was found compatible with the Laryngeal Mask AuraGain™ Size 3 insertion for both standard and rotational LMA insertion techniques.

### 2.3. Study Design and Sample Size

**Figure 1:**
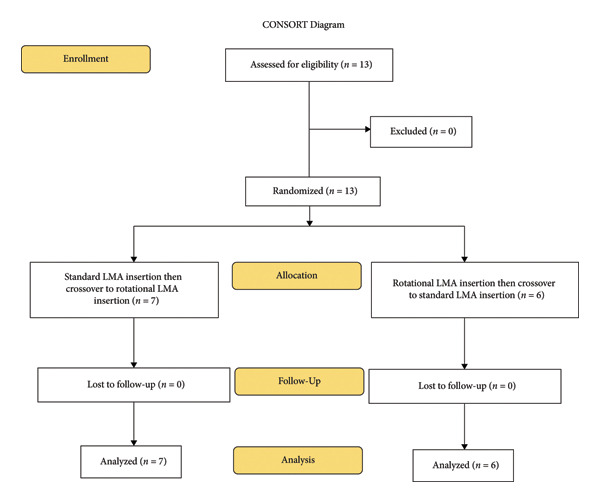
Consolidated Standards of Reporting Trials (CONSORT) flow diagram defining trainees’ enrollment in the present study.

The study design was a randomized controlled crossover trial. Written informed consent was obtained from study participants. The trainees were allowed to insert the LMA 10 times each with standard and rotational insertion techniques in a crossover manner by the division of two groups with the draw method. The one study group has initiated the LMA insertion with the standard insertion technique, and the other group has started with the rotational insertion technique; afterward, the groups crossed over the insertion techniques (Figure 1).

The sample size estimation was based on a pilot study performed on a manikin. Three novice anesthesia trainees had performed 10 LMA insertion attempts with the standard technique and 10 LMA insertion attempts with the rotational technique for LMA insertion ease (successful insertion attempts and insertion time duration). The author discovered that 60% of LMA insertion attempts were successful within 10–20 s and 20% within 20–30 s, and the remaining 20% required more than 30‐s time duration. Mean ± SD insertion times were 10 ± 6.4 s for the standard technique and 12 ± 7.6 s for the rotational technique. Using a two‐sided test with a 5% significance level (*α* = 0.05) and 80% power, the required sample size was 97 LMA insertion attempts with each study insertion technique. It was rounded up to a total of 200 LMA insertion attempts with both standard and rotational LMA insertion techniques.

Based on the pilot study and to align with the department’s annual resident recruitment cycle, the 10 trainees were required to target 200 LMA insertion attempts; each trainee performed 10 LMA insertion attempts with the standard LMA insertion technique and 10 LMA insertion attempts with the rotational LMA insertion technique. However, each trainee was supposed to perform 20 LMA insertion attempts, and the sample size calculation was based on the assumption of independence between the insertion attempts. Moreover, it was supported with the defined washout period and ensured the absence of learning effects on the study’s outcomes. The author has used a similar methodology to test the two different techniques of i‐gel insertion among novice anesthesia trainees [16].

### 2.4. Study Protocol

The eligible study participants have received the detailed information at the time of enrollment. Following the questionnaire‐based evaluation of prior knowledge for SADs, insertion techniques, and insertion experiences, the study started with an educational tutorial of Brian’s standard LMA insertion technique and rotational LMA insertion technique. In the second phase, the participants were shown the video presentation of both studies’ insertion techniques. Afterward, the trainees were demonstrated practical conduct of Brian’s standard LMA insertion technique [1] (the LMA was held like a pen, and the index finger was placed at the junction of the LMA tube and cuff. The index finger was used to press LMA against the hard palate and to the posterior pharyngeal wall until definite resistance was felt at the base of the hypopharynx. Then, LMA was held with the nondominant hand and the index finger was removed at its final placement position) and rotational LMA insertion technique [1] (LMA was inserted and placed like the guedel airway insertion. The LMA was proximally grasped close to the side of the anesthesia circuit attachment. Afterward, the LMA insertion began with the LMA cuff facing toward the nose and hard palate, and then, it was advanced up to the base of the hypopharynx or until resistance was felt. At this point, the LMA was rotated at 180°, anticlockwise, and the LMA tube black line was positioned and confirmed on the nasal side), on a manikin by the study’s investigators. The manufacturer’s instructions were followed such that the manikin’s oral cavity was lubricated with water‐based jelly, and the deflated Size 3 LMA was also lubricated for practice sessions and for data collection. The successful LMA insertion was considered on the manikin’s chest (lungs) inflation with Ambu bag insufflation of sufficient manikin chest movement. The study’s participants were allowed sufficient time to practice both standard and rotational LMA insertion techniques on a manikin. The failure of LMA insertion attempts was labeled as the trainees being unable to insert the LMA or failing to insufflate the manikin’s lungs.

The primary outcome of the study was recorded by experienced anesthesiologists for each LMA insertion attempt and time duration; each trainee performed 10 consecutive LMA insertion attempts with standard and rotational LMA insertion techniques. The time duration of LMA insertion was counted from the holding of the LMA till the confirmation of the manikin chest rise. The secondary outcome of the study was questionnaire‐based, and participants were asked at the start of the study about the correlation of prior knowledge of SADs, LMA insertion techniques, and LMA insertion experiences. The tertiary outcome of the study was also questionnaire‐based, and participants were interviewed at the end of the study for the preferred LMA insertion technique, on the basis of learning method and practical adaptability (user‐friendly) for the novice anesthesia trainees.

### 2.5. Data Analysis

Microsoft Excel for Mac (version 16.26; Microsoft Corp., Redmond, WA, USA) and Stata version 16 were used to analyze the data and to make the cumulative sum (CUSUM) chart. Mean and standard deviation were computed for the quantitative variables, such as age and LMA insertion time duration. Frequency and percentage were computed for gender, prior knowledge of SADs, previous work experience with airway management, and trainees’ preference for practical insertion technique. CUSUM analysis was performed according to the time duration of device placement such as 10–20 s, 20–30 s, and greater than 30 s. An acceptable failure rate is set at 20% (*p*_0_ = 0.2), and an unacceptable failure rate is set at 40% (*p*_1_ = 0.4). Also, we set the Type I error rate = 0.1 and the Type II error rate = 0.1. The values of *p*0, *p*1, *a*, and *b* were determined for time duration (s) by which the sum decreases if there is a “success,” and in case of “failure,” the sum increases by 1 s. The CUSUM chart is completed by upper (*h*_1_) and lower (*h*_0_) boundary lines. The distance between two adjacent boundary lines depends on the probability of a Type I error (risk of declaring competence when it is not achieved) and a Type II error (risk of not declaring competence when it has been attained). A line to describe the progress of each resident was drawn on the basis of the CUSUM chart. The resident was considered “proficient” if his/her line crossed the two adjacent or consecutive boundary lines from above; that is, an acceptable failure rate will be reached. The resident will be deemed “not proficient” and in need of further training if the graphical trend is upward or if it moves downward at each attempt but fails to cross two consecutive boundary lines from above. The comparison of the learning effect of residents for both standard LMA and rotational LMA insertion techniques was observed on the CUSUM chart. The *p*‐value of ≤ 0.05 was considered statistically significant.

## 3. Results

**Figure 2:**
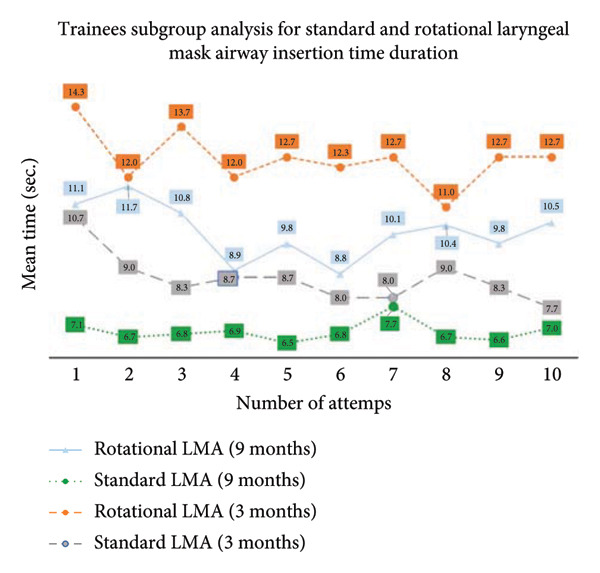
Trainees’ subgroup analysis for time duration of standard and rotational laryngeal mask airway insertions.

The study was planned to test 10 participants. However, the 13 novice anesthesia trainees from multicenter hospitals had accepted and participated in our study. The trainees’ age was unremarkable, but the number of female trainees was significant compared to the male trainees. Another variation was noticeable in the training startup duration such as 10 trainees who had started the anesthesia training 9 months ago and three trainees who had started 3 months ago (Table 1). The trainees’ prior knowledge of the type of SADs and practice knowledge were equivocal (Table 1). All 13 trainees were aware of the standard LMA insertion technique compared to 10 trainees who had shown awareness of the rotational LMA insertion technique. Further subgroup analysis demonstrated the prior experience with the standard LMA insertion technique; three trainees had less than 10 times of insertion experience, and 10 trainees had more than 21 times of insertion experience (Table 1). For the rotational LMA insertion technique, the nine trainees have shown prior experience of less than 10 times insertions, and one trainee had stated having less than 20 times insertions (Table 1). The study revealed that participants in both study groups showed a time duration of 7.92 ± 2.43 s on the first attempt with standard LMA insertion, compared to 7.15 ± 1.72 s on the tenth attempt with standard LMA insertion (Table 2). The first attempt with the rotational LMA insertion technique was 11.80 ± 3.41 s, compared to 11.00 ± 2.89 s on the tenth rotational LMA attempt on manikin (Table 2). Nevertheless, the time duration of the standard and rotational LMA insertions gradually shortened with each rising insertion attempt, but it was not at the significant level. Moreover, the crossover (study participants separated into two groups) analysis also revealed a lesser time duration with the standard LMA insertion technique compared to the rotational LMA insertion technique, whether trainees began standard LMA insertion before rotational LMA insertion (Table 3) or trainees began rotational LMA insertion before standard LMA insertion (Table 4) on manikin. Furthermore, the trainees’ startup variability analysis showed a huge impact of 9 months and 3 months of anesthesia training experience on the success of LMA insertion time duration. The trainees with 9 months of experience performed the standard LMA insertion in 7.1 s, compared to 10.7 s among trainees with 3 months of experience; similarly, the trainees with 9 months of experience performed the rotational LMA insertion in 11.1 s compared to 14.3 s among trainees with 3 months of experience (Figure 2). However, the *p*‐value was consistently significant between standard and rotational LMA insertion techniques (Table 2). In contrast, the CUSUM analysis did not demonstrate the competency with each rising LMA insertion attempt in both standard and rotational LMA insertion techniques (Figures 3 a and b). The post–study analysis revealed the standard LMA insertion technique is easy to learn on manikin by 12 trainees, and 11 trainees have shown a preference to practice at the beginning of anesthesia training (Table 5). Nevertheless, the nine trainees have elicited interest for the rotational LMA insertion along with the standard LMA insertion to practice most of the time in their practice.

**Table 1:** Trainees’ demographics, prior supraglottic airway device (SAD) information, and practical laryngeal mask airway insertion experience.

Variables	Both study groups
Age (years [mean ± SD])	28.4 ± 3.23
Gender [female/male]	11 [84.6%]/2 [15.4%]
Anesthesia training duration	
3 months/9 months	3 [23.08%]/10 [76.92%]
Prior SAD information	
LMA	13 [100%]
i‐gel	11 [84.6%]
ProSeal LMA	2 [15.38%]
Other	1 [7.69%]
Prior SAD insertion technique information	
LMA	13 [100%]
i‐gel	11 [84.6%]
ProSeal LMA	2 [15.38%]
Intubating LMA	1 [7.69%]
Prior standard LMA insertion technique information	13 [100%]
Prior rotational LMA insertion technique information	10 [76.92%]
Prior standard LMA insertion experience (number of attempts [mean ± SD])	[31.8 ± 25.8]
0–10	3
11–20	0
21 and above	10
Prior rotational LMA insertion experience	
(number of attempts [mean ± SD])	[2.77 ± 4.42]
0–10	9
11–20	1
21 and above	0

*Note:* Data presented as mean ± SD or N [%] or percentage.

**Table 2:** Number of LMA insertion attempts with standard and rotational techniques on manikin.

Number of LMA insertion attempts	Standard technique	Rotational technique	*p*‐Value
1	7.92 ± 2.43	11.80 ± 3.41	< 0.001
2	7.23 ± 2.05	11.80 ± 3.85	< 0.001
3	7.15 ± 1.57	11.50 ± 3.67	0.002
4	7.31 ± 2.02	9.62 ± 3.52	0.003
5	7.00 ± 2.08	10.50 ± 5.14	0.012
6	7.08 ± 1.80	9.62 ± 3.52	0.004
7	7.77 ± 1.54	10.70 ± 2.95	< 0.001
8	7.23 ± 2.17	10.50 ± 2.85	0.001
9	7.00 ± 1.91	10.50 ± 3.64	0.001
10	7.15 ± 1.72	11.00 ± 2.89	0.001

*Note:* Data presented as mean ± SD. *p*‐Values significant at < 0.05.

**Table 3:** Seven study participants began with the standard LMA insertion technique before rotational LMA insertion in manikin.

Number of LMA insertion attempts	Standard technique	Rotational technique	*p*‐Value
1	7.00 ± 0.63	10.70 ± 2.34	0.010
2	6.67 ± 1.03	12.30 ± 4.80	0.036
3	7.00 ± 1.41	11.00 ± 2.34	0.041
4	7.50 ± 1.05	8.83 ± 1.17	0.010
5	6.50 ± 0.84	11.20 ± 3.40	0.044
6	7.17 ± 0.75	9.33 ± 2.10	0.038
7	7.17 ± 1.17	10.50 ± 2.51	0.019
8	7.00 ± 1.41	10.20 ± 2.13	0.040
9	6.67 ± 1.37	11.20 ± 3.06	0.032
10	6.67 ± 1.03	10.80 ± 2.48	0.015

*Note:* Data presented as mean ± SD. *p*‐Values significant at < 0.05.

**Table 4:** Six study participants began with rotational LMA insertion before standard LMA insertion in manikin.

Number of LMA insertion attempts	Rotational technique	Standard technique	*p*‐Value
1	8.71 ± 3.15	12.90 ± 4.02	0.002
2	7.71 ± 2.63	11.30 ± 3.15	0.001
3	7.29 ± 1.80	11.90 ± 3.89	0.008
4	7.14 ± 2.67	10.30 ± 4.35	0.024
5	7.43 ± 2.76	9.86 ± 3.98	0.039
6	7.00 ± 2.45	9.86 ± 4.41	0.038
7	8.29 ± 1.70	10.90 ± 3.48	0.034
8	7.43 ± 2.76	10.90 ± 2.79	0.001
9	7.29 ± 2.36	9.86 ± 4.22	0.017
10	7.57 ± 2.15	11.10 ± 3.39	0.012

*Note:* Data presented as mean ± SD. *p*‐Values significant at < 0.05.

Figure 3(a) CUSUM sum of successful standard LMA insertion attempts. (b) CUSUM sum of successful rotational LMA insertion attempts.

**Figure:**
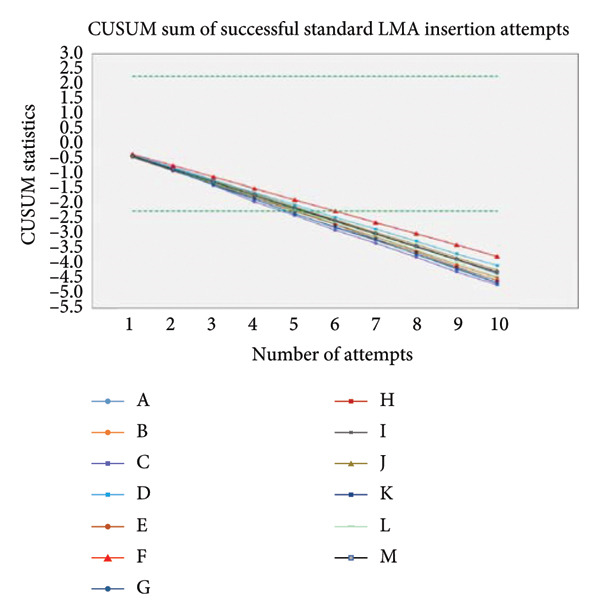
(a)

**Figure:**
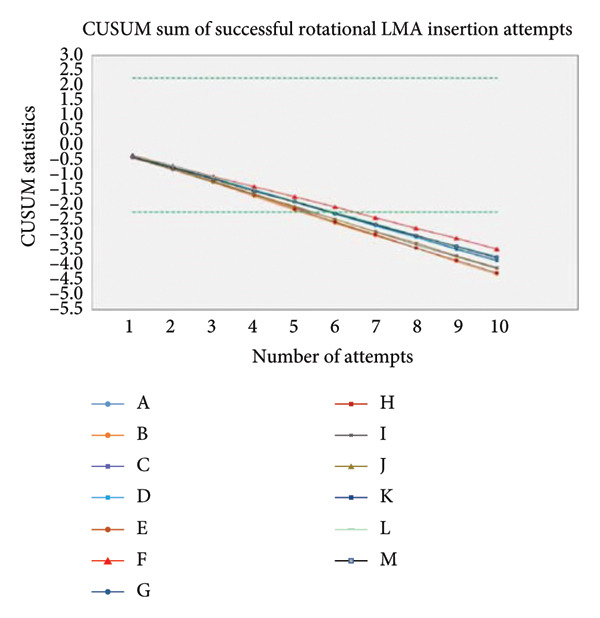
(b)

**Table 5:** Trainees’ post–study priority learning and practical preference for LMA insertion techniques.

Metrics	Standard technique	Rotational technique	*p*‐Value
LMA insertion technique easy to learn at the start of anesthesia training	12 (92.30%)	1 (7.7%)	< 0.001
LMA insertion technique practical preference at the start of anesthesia training	11 (84.6%)	2 (15.4%)	< 0.001
LMA insertion technique preference to practice at most of the time	13 (100%)	9 (69.2%)	0.030

*Note:* Data presented as N (%) or percentage. *p*‐Values significant at < 0.05.

## 4. Discussion

This randomized controlled crossover trial study found the standard LMA insertion technique is relatively easy to learn and practice on manikin by novice anesthesia trainees compared to the rotational LMA insertion technique. The failure attempts with both studies’ LMA insertion technique were zero. The significance noticeable in the time duration for the first attempt with the standard LMA insertion technique was 7.92 ± 2.43 s, compared to the first attempt with the rotational LMA insertion technique of 11.80 ± 3.41 s. Moreover, the study found the time duration of all 10 consecutive standard LMA insertion attempts was shorter than the time duration of consecutive rotational insertion attempts. Our study recruited multicenter Year 1 anesthesia trainees, and it found variations in anesthesia training startup months. Although all trainees had successfully inserted an LMA in manikins with both studies’ insertion techniques, trainees with 9 months of experience cited relatively better ease compared to trainees with 3 months of training experience. The CUSUM analysis was designed to monitor the proficiency with each rising LMA insertion attempt with both studies’ insertion techniques. Nevertheless, the CUSUM plotting variations were found to be insignificant among the trainees with succeeding LMA insertion attempts. Furthermore, the crossover maneuver was applied to discover the trainees’ learning preference for the LMA insertion technique either to begin with the standard or rotational technique at the early phase of anesthesia training. The trainees were reckoned for the standard LMA insertion technique. It is easy to learn and practice on manikin either before or after the commencement of the rotational LMA insertion technique experience. Moreover, the study participants’ prior knowledge of type of supraglottic devices and insertion techniques appeared proportionately analogous to the anesthesia training months. The post–study analysis for the priority LMA insertion technique has favored the first preference for the standard LMA insertion technique, followed by the rotational technique in clinical practice.

The simulation‐based manikin studies are allowing the academician and researcher to train and test the airway skills in a controlled setting [17]. The LMA has been studied in manikin for insertion techniques, device feasibilities, and ventilation accuracy and to simulate emergency airway management. Our study used Laerdal SimMan (Laerdal Medical AS, AUS), manikin, and Laryngeal Mask AuraGain™ Size 3, for both standard and rotational LMA insertion techniques.

The literature is extensively immersed with repetitive unsuccessful LMA insertion attempts with Brian’s recommended LMA insertion technique in elective and emergency case scenarios [18]. In our study, both the standard and rotational LMA insertion techniques achieved a 100% first‐attempt success rate, along with a 100% success across all 10 consecutive LMA insertion attempts with both standard and rotational techniques on manikin. This aligns with the findings of Lee et al. [12], who had reported a 100% success rate for standard LMA insertion in a manikin‐based study. In contrast, Stroumpoulis et al. [19] reported a significantly lower success rate of 63.8% with a similar simulation technique. In addition, the human studies have reported lower first attempts with standard LMA insertion rates such as 83.3% [14], 84.3% [15], and 86% [8]. This study will be the first to evaluate the rotational LMA insertion technique on manikin, and it has discovered a 100% success rate on the first attempt and a 100% success rate across 10 consecutive insertion attempts on manikin. Nevertheless, the rotational LMA insertion technique has been extensively studied in human studies. The reported first‐attempt success rate varies such as 86% [8, 15] and 93.5% [14] in adults and up to 99% [20] in pediatric patients.

Additionally, our study demonstrated that the mean time duration for the first attempt with the standard LMA insertion technique was 7.92 ± 2.43 s, which is significantly shorter compared to the manikin‐based studies among experienced physicians (13.9–19.3 s [12] and 13.64 ± 6.55 s [18]) and novice physicians (22.0 ± 4.41 s) [18]. Furthermore, the human studies have reported even greater time durations for first‐attempt LMA insertion such as 30.26 ± 4.84 s [15] and 37.68 ± 12.42 s [14]. Similarly, rotational LMA insertion time duration (11.80 ± 3.41 s) in our manikin study is significantly shorter than those reported in human studies (39.03 ± 14.12 s [14] and 32.77 ± 4.08 s) [15], although it has to be compared with manikin to manikin. Nevertheless, the reduced standard LMA insertion duration in our study has multifactorial variants like our study participants who were anesthesia trainees with previous knowledge, who received structured orientation along with practice on manikin compared to the published studies’ participants, and nonanesthesiologist physicians and paramedical personnel.

The supraglottic airway insertion proficiency is reported in some studies, like fewer than 10 insertion attempts required in one study [21] and up to 15 insertion attempts indicated in another study that applied the CUSUM analysis [22]. In contrast, our study evaluated 10 consecutive insertion attempts per participant, and overall, it was the 20 insertion attempts with both studies’ insertion techniques.

Our study limitations are as follows: First, the use of a manikin model limits the generalizability of our findings to clinical practice because manikins do not accurately replicate important clinical variables such as LMA leak pressure, lung inflation tidal volume, or the potential for complications such as insertion trauma and postoperative sore throat. Second, our study has used LMA‐AuraGain, and the comparative analysis studies have used classic and other LMA variants, so the comparison may not be truly reflective. Third, there was variability in the training duration among participants, although this difference did not reach statistical significance in our analysis. Fourth, our study has recruited a restricted number of participants, although the total LMA insertions were adequate in both groups. This may have affected the generalizability of the results. Fifth, our study was unable to compare the rotational LMA insertion among manikin studies.

## 5. Conclusion

Our study concludes the standard LMA insertion technique is relatively easy to learn and practice on manikin among novice anesthesia trainees compared to the rotational LMA insertion technique. Additionally, the novice anesthesia trainees have vouched for standard LMA insertion as a priority technique in their clinical practice. Nevertheless, the manikin simulation study cannot mimic the real patient experience, when considering the airway anatomy and tissue responsiveness.

## Ethics Statement

This randomized controlled crossover trial was approved by the Research Ethical Review Committee IRB (2021‐62657‐21893), Aga Khan University Hospital, Karachi, Pakistan.

## Consent

The written informed consent was obtained from all the study participants.

## Conflicts of Interest

The authors declare no conflicts of interest.

## Author Contributions

Kumar designed the study and wrote the manuscript. Kala did a literature search. Both authors performed the study, collected the data, gathered the data, and analyzed the data.

## Funding

No funding was required for this trial.

## Data Availability

Data are available on request from the authors.
